# Mental health treatment among older adults with mental illness on parole or probation

**DOI:** 10.1186/s40352-019-0084-y

**Published:** 2019-03-28

**Authors:** William C. Bryson, Brandi P. Cotton, Lisa C. Barry, Martha L. Bruce, Jennifer Piel, Stephen M. Thielke, Brie A. Williams

**Affiliations:** 1 Oregon Health & Science University, 3181 SW Sam Jackson Park Road, Multnomah Pavilion, Room 2316, Portland, OR 97239-3098 USA; 2 University of Rhode Island, College of Nursing, Kingston, RI USA; 3 UConn Center on Aging, University of Connecticut School of Medicine, Farmington, CT USA; 4 Geisel School of Medicine, Dartmouth University, Hanover, NH USA; 5 University of Washington Medical Center, Seattle, WA USA; 6 Puget Sound Veterans Affairs Medical Center, Seattle, WA USA; 7 Division of Geriatrics, University of California San Francisco, San Francisco, CA USA

**Keywords:** Parole, Probation, Mental illness, Mental health treatment, Older adults

## Abstract

**Background:**

The number of older adults on parole and probation is growing at an unprecedented rate, yet little is known about the mental health needs and treatment utilization patterns among this group. The objective of this study is to compare the prevalence of serious or moderate mental illness (SMMI), and the proportion of those with SMMI who receive mental health treatment, among community-dwelling older adults on correctional supervision (parole or probation) vs. not on correctional supervision.

**Methods:**

*Design:* Cross-sectional analysis of data from the 2008–2014 National Surveys for Drug Use and Health (NSDUH).

*Setting:* Population-based national survey data.

*Participants:* Older adults (age ≥ 50) who participated in the NSDUH between 2008 and 2014 (*n* = 44,624). Participants were categorized according to whether they were on parole or probation during the 12 months prior to survey completion (*n* = 379) vs. not (*n* = 44,245).

*Measurements:* Probable SMMI was defined using a validated measure in the NSDUH. Mental health treatment included any outpatient mental health services or prescriptions over the 12 months prior to survey completion. We compared the prevalence of SMMI, and the proportion of those with SMMI who received any treatment, by correctional status.

**Results:**

Overall, 7% (*N* = 3266) of participants had SMMI; the prevalence was disproportionately higher among those on parole or probation (21% vs. 7%, *p* <  0.001). Sixty-two percent of those with SMMI received any mental health treatment, including 81% of those on parole or probation and 61% of those who were not (*p* <  0.001). This result remained statistically significant after logistic regression accounted for differences in sociodemographics and health.

**Conclusions:**

SMMI is disproportionally prevalent among older adults on parole or probation, and community correctional supervision programs may be facilitating linkages to needed community-based mental health treatment.

## Introduction

Community correctional programs, such as parole and probation, form an important coordinating bridge between the criminal justice and community healthcare systems for individuals with mental illness (Epperson et al., 2014; Lamberti, 2016; Morrissey, Fagan, & Cocozza, 2009; Munetz & Griffin, 2006; Osher & King, 2015), but little is known about their role in mental health services coordination for older adults (Maschi, Sutfin, & O’Connell, 2012). This is a critical knowledge gap, since older adults (those in their 50s or older) are the fastest growing age demographic in prisons and jails (Carson & Anderson, 2016; Carson & Sabol, 2016), there are up to four times as many older adults on community correctional supervision as there are incarcerated (McCarthy & Langworthy, 1987), and their burden of mental health needs is disproportionate to the general population of older adults (Bryson, Cotton, & Brooks, 2017).

Mental health-related conditions are more prevalent among older adults on parole or probation than older adults without justice involvement, including past-year major depression (15% vs. 5%), serious psychological distress (22% vs. 6%), alcohol use disorders (20% vs. 3%), and drug use disorders (7% vs. 0.6%) (Bryson et al., 2017). Advanced age and mental illness have been independently linked to poor social supports, severe material deprivation, and problems with social reintegration upon release from incarceration (Dobmeier et al., 2017; Western, Braga, Davis, & Sirois, 2015; Wyse, 2018). The combination of advancing age and mental illness may define a subgroup of justice-involved individuals with exceptional difficulty following release from incarceration, including a profound need for structured support and mental health services, and significant barriers to healthcare access. Yet, while it is known that the need for mental health treatment is common in this population, it is unknown whether treatment is received.

Therefore, in this study, we determined the prevalence of serious or moderate mental illness (SMMI) among older adults on parole or probation, and assessed the percentage of those with SMMI who received any mental health treatment over the course of 1 year. We compared the prevalence and treatment of SMMI among older adults on parole or probation to those among older adults who were not on community corrections. In light of prior research suggesting considerable barriers to healthcare access among older justice-involved individuals (Bryson et al., 2017; Western et al., 2015), our primary hypothesis was that a smaller proportion of older adults with SMMI in the justice-involved group would receive treatment.

## Methods

### Participants and procedures

In this cross-sectional study, we analyzed data from the National Survey on Drug Use and Health (NSDUH). The NSDUH is an annual survey sponsored by the Substance Abuse and Mental Health Services Administration (SAMHSA) to measure the prevalence and correlates of drug use (https://www.samhsa.gov/data/data-we-collect/nsduh-national-survey-drug-use-and-health). The survey employs an independent, multistage probability sample design, and is conducted through a combination of audio computer-assisted self-interview and computer-assisted personal interview. Participants are non-institutionalized individuals aged 12 or older. People residing in institutions are excluded (including those confined to jails, prisons, nursing homes, or hospitals). Homeless people not staying in shelters are also excluded. There are no exclusions based on history of incarceration. Participants receive $30 for their participation.

We pooled data from seven consecutive NSDUH surveys from 2008 to 2014, and restricted our analyses to respondents aged 50 or older. This cutoff is in line with other studies, which – due to the premature development of illness and functional impairment in many justice-involved older adults (often referred to as “accelerated aging”) – define the threshold for “older adult” in this population to begin at age 50 or 55 (Aday, 2003; Williams, Goodwin, Baillargeon, Ahalt, & Walter, 2012).

### Measures

#### Criminal justice supervision status

We categorized those on parole or probation as being under community criminal justice supervision. Parole refers to a period of conditional supervised release in the community following a prison term. Probation is a court-ordered period of community correctional supervision, often used as an alternative to incarceration. The NSDUH contains two yes/no questions about parole and probation status: “Were you on probation at any time during the past 12 months?” and “Were you on parole, supervised release, or other conditional release from prison at any time during the past 12 months?” Responses were classified into groups with and without any community correctional supervision over the past year.

#### Serious or moderate mental illness (SMMI)

The NSDUH data include dichotomous (yes/no) indicators of mild, moderate, and serious mental illness. Developed and validated by the Substance Abuse and Mental Health Services Administration (SAMHSA) and the National Institute of Mental Health (NIMH), these indicators predict mental illness based upon responses to several items in the NSDUH questionnaire (Center for Behavioral Health Statistics and Quality, 2015a). The items used to identify mental illness include level of emotional distress, functional impairment due to emotional distress, suicidal thoughts, and major depression. Prior research has used these NSDUH items to define a population with mental health conditions (Han, Compton, Mojtabai, Colpe, & Hughes, 2016). Our definition of SMMI included positive indicators for serious or moderate mental illness, which is equivalent to Global Assessment of Functioning (GAF) scores of < 60 (serious mental illness is GAF < 50; moderate mental illness is 50 ≤ GAF < 60) (Center for Behavioral Health Statistics and Quality, 2015b). Individuals with mild mental illness, defined as GAF ≥ 60, were not included, as their mental health treatment needs are likely to be different (Kessler et al., 2003).

#### Mental health treatment

We defined mental health treatment as any outpatient mental health visits or prescriptions in the past 12 months. We included prescription medications from all prescribers in our definition of mental health treatment, consistent with prior studies suggesting that older adults often receive mental health prescriptions from their primary care physician and not mental health providers (Barry, Abou, Simen, & Gill, 2012). We categorized the absence of any outpatient visits or prescriptions, in the setting of SMMI, as “untreated” mental illness.

Outpatient visits were determined by a response of yes or no to the question: “During the past 12 months, did you receive any outpatient treatment or counseling for any problem you were having with your emotions, nerves, or mental health at any of the places listed below? Please do not include treatment for alcohol or drug use.” The places listed were: an outpatient mental health clinic or center; an outpatient medical clinic; the private office of a therapist, psychologist, psychiatrist, medical doctor, social worker, or counselor; a partial hospitalization or day treatment program; or, “some other place.” Prisons and jails were not listed among the options, so treatment delivered in those settings was not likely to be captured in this measure. Prescriptions were determined by the following yes/no question: “During the past 12 months, did you take any prescription medication that was prescribed for you to treat a mental or emotional condition?”

#### Sociodemographics and health

We assessed demographic, socioeconomic, and clinical covariates. Demographic variables included age (50–64 vs. ≥65), gender, and race/ethnicity (black, white, Hispanic/Latino). Socioeconomic variables included marital status, educational attainment (≥high school graduation), full-time or part-time employment, income poverty (household income below the federal poverty limit), and any health insurance over the past year. Self-rated health (poor/fair vs. good or better) was included to describe health status.

Substance use disorders were defined based upon diagnostic criteria for substance abuse and dependence in the *Diagnostic and Statistical Manual of Mental Disorders, 4th Edition (DSM-IV)*. Numerical values of the Kessler-6 Psychological Distress Scale (K6) score for the worst month of the past year were used as a continuous measure of psychological distress, since differences in symptom severity could affect the association between justice involvement and mental health treatment (Kessler et al., 2003).

### Statistical analysis

We compared the overall characteristics of the two groups (those on community correctional supervision vs. all others) using student’s t-tests, Chi-Squared tests, and Fisher’s exact tests when cell sizes were small (5 or fewer). We then calculated the proportion of individuals with SMMI in each group who received any mental health treatment over one year, and stratified the results by each covariate to examine for potential interactions. Finally, we developed a multivariate logistic regression model to investigate the association between parole or probation status and mental health treatment among older adults with SMMI, while adjusting for all covariates and survey year (to account for changes in health insurance legislation from 2008 to 2014).

Due to multiple comparisons, we highlighted both *p*-values < 0.01 and <  0.001. Records with any elements missing were excluded (*n* = 10 on parole or probation, and *n* = 142 not on community supervision). Our analyses did not incorporate survey weights or sampling characteristics because the stratified sample sizes in the parole or probation group were too small to produce reliable nationally representative prevalence estimates (Center for Behavioral Health Statistics and Quality, 2015a).

All data that we analyzed are publically available and de-identified, and therefore did not constitute human subjects research according to the institutional review board at the University of Washington. All statistical analyses were performed with STATA software version 13.1 (STATA Corporation, College Station, TX).

## Results

**Fig. 1:**
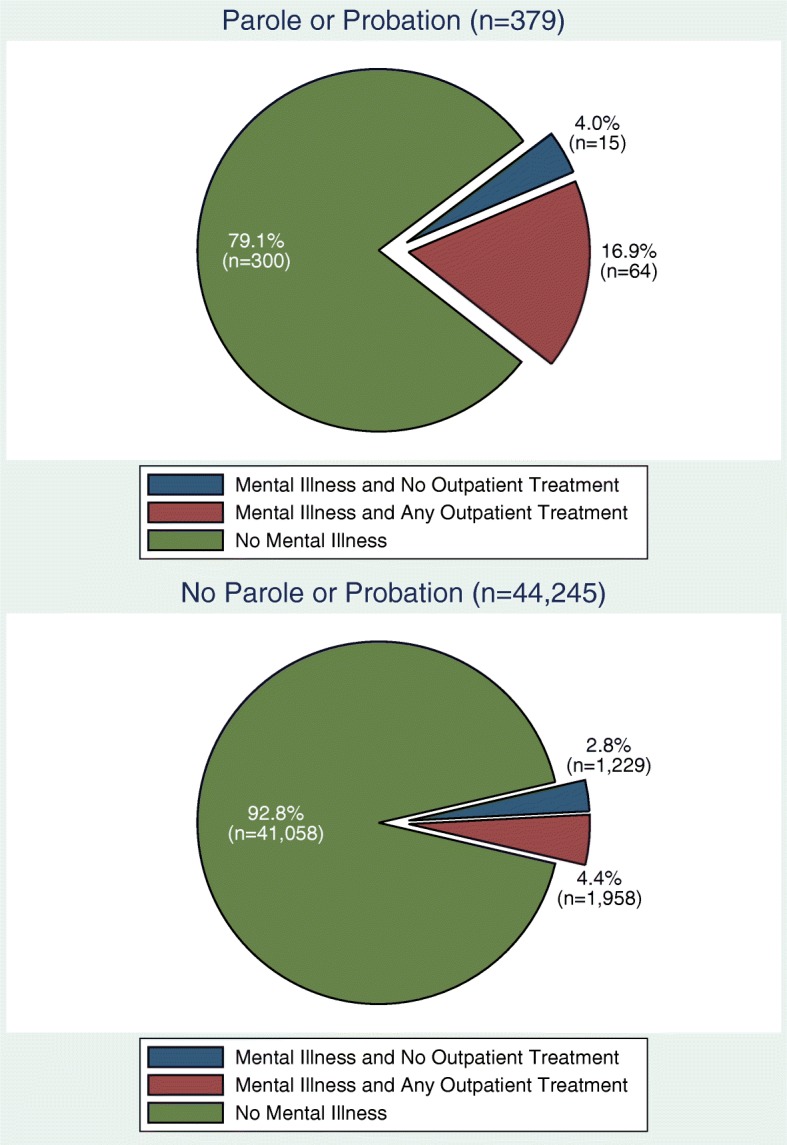
Prevalence of Serious or Moderate Mental Illness (SMMI) and Use of Outpatient Mental Health Services

Overall, 44,624 NSDUH survey responders aged 50 or older completed questionnaires in 2008–2014: 7.3% (*n* = 3266) reported having serious (*n* = 1453) or moderate (*n* = 1813) mental illness (SMMI); 0.8% (*n* = 379) reported being on parole or probation over the past year. SMMI was reported among 20.9% (*n* = 79) of older adults on parole or probation, and 7.2% (*n* = 3187) of those not on community supervision (*p* <  0.001; see Fig. 1).

### Participant characteristics

Among those older individuals with SMMI (n = 3266), several characteristics differed according to community correctional supervision status (Table 1). Those on parole or probation were younger than those not on community correctional supervision (8% vs. 22% aged 65 or older), and fewer were female (46% vs. 67%). Those on community supervision had more socioeconomic disadvantages: 23% vs. 46% were married; 66% vs. 83% had graduated high school; and 39% vs. 21% had a household income below the federal poverty level. They were also more likely to have a co-occurring substance use disorder (41% vs. 11%), and their psychological distress was higher (mean K6 score 16.3 vs. 14.6).

**Table 1:** Descriptive statistics

	Full Sample		Subset With Serious or Moderate Mental Illness				
			Parole or Probation in the Past 12 Months				
			No		Yes		
Characteristics	n	%	n	%	n	%	*p*-value
Total	44,624	100	3187	100	79	100	–
Parole or Probation	379	0.9	0	0	79	100	–
Mental Illness							
Serious	1453	3.3	1409	44.2	44	55.7	0.042
Moderate	1813	4.0	1778	55.8	35	44.3	0.042
Either	3266	7.3	3187	100.0	79	100.0	–
Age 65 and over	17,102	38.3	698	21.9*	6	7.6*	0.002
Female	24,501	54.9	2121	66.6**	36	45.6**	< 0.001
Black	4521	10.1	280	8.8	13	16.5	0.018
Hispanic	3824	8.6	252	7.9	8	10.1	0.472
Married	26,934	60.4	1471	46.2**	18	22.8**	< 0.001
Graduated High School	37,549	84.2	2638	82.8**	52	65.8**	< 0.001
Employed	22,300	50.0	1151	36.1	19	24.1	0.027
Income Poverty	4583	10.3	674	21.2**	31	39.2**	< 0.001
Health Insurance	41,325	92.6	2863	89.8	64	81.0	0.011
Substance Use Disorder	1846	4.1	364	11.4**	32	40.5**	< 0.001
Self-Rated Health Poor or Fair	9270	20.8	1486	46.6	38	48.1	0.795
	Mean	S.D.	Mean	S.D.	Mean	S.D.	*p*-value
Kessler-6 Score for Worst Month of the Past Year	3.98	4.79	14.56*	5.02	16.25*	5.19	0.003

* *p* <  0.01; ** *p* <  0.001

### Outpatient mental health treatment: descriptive results

Among the subset of older adults who had SMMI, the proportion that received any mental health treatment was 20% higher among those with recent parole or probation compared to those without community correctional supervision (81% vs. 61% received any treatment, *p* <  0.001; Table 2). This difference was reduced among individuals aged 65 and over (50% vs. 50%), and those with no health insurance (53% vs. 51%). The difference in mental health treatment was larger for individuals of black race (85% on community corrections vs. 48%; *p* = 0.011). However, given the small number of older adults on parole or probation with no health insurance (*n* = 15), Black race (*n* = 13), or age 65 or older (*n* = 6), these potential interactions must be interpreted with caution.

**Table 2:** Proportion of Older Individuals With SMMI Who Received Outpatient Mental Health Treatment: Descriptive Results

Characteristic		Proportion of Individuals With SMMI Who Received Any Treatment (%)		*p*-value^a^
		Parole or Probation		
		No	Yes	
Total		61.4**	81.0**	< 0.001
Age	50–64	64.5**	83.6**	< 0.001
	65 or over	50.4	50.0	1.000 (F)
Gender	Male	55.6*	79.1*	0.002
	Female	64.4	83.3	0.018
Black Race	No	62.7*	80.3*	0.003
	Yes	48.2	84.6	0.011 (F)
Hispanic Ethnicity	No	62.4**	81.7**	< 0.001
	Yes	50.4	75.0	0.282 (F)
Married	No	61.8**	83.6**	< 0.001
	Yes	61.1	72.2	0.334
Graduated High School	No	51.7	74.1	0.023
	Yes	63.5*	84.6*	0.002
Employed	No	65.0*	83.3*	0.003
	Yes	55.1	73.7	0.106
Income Poverty	No	61.2	79.2	0.011
	Yes	62.3	83.9	0.015
Health Insurance	No	51.5	53.3	0.892
	Yes	62.6**	87.5**	< 0.001
Substance Use Disorder	No	61.2	78.7	0.014
	Yes	63.2	84.4	0.016
Poor or Fair Self-Rated Health	No	60.7	78.1	0.025
	Yes	62.3*	84.2*	0.006

* *p* < 0.01; ** *p* < 0.001

^a^(F) = Fisher’s exact test used. Otherwise, Chi-Squared tests were used

### Outpatient mental health treatment

After adjusting for differences in demographic, socioeconomic, and clinical characteristics, the proportion of individuals with SMMI who received any mental health treatment remained 20% higher in older adults on parole or probation compared to those not on community correctional supervision (adjusted OR: 2.85; Table 3). Other characteristics that were associated with mental health treatment included female gender (adjusted OR: 1.41), education ≥high school graduation (adjusted OR: 1.61), health insurance (adjusted OR: 2.05), and psychological distress (adjusted OR: 1.06 for each additional point on the K6 scale). Characteristics that were inversely associated with mental health treatment included age 65 or older (adjusted OR: 0.44), black race (adjusted OR: 0.55), Hispanic/Latino ethnicity (adjusted OR: 0.57), and employment (adjusted OR: 0.49).

**Table 3:** Proportion of Older Individuals with SMMI who Received Outpatient Mental Health Treatment: Logistic Regression Results

Characteristic		Proportion of Individuals With SMMI Who Received Any Treatment (%)	Unadjusted Difference (%) (95% CI)	Adjusted Difference (%) (95% CI)	Adjusted Odds Ratio (95% CI)
Parole or Probation	No (reference)	61.4	19.6** (10.8, 28.4)	19.3** (10.4, 28.1)	2.85** (1.56, 5.21)
	Yes	81.0			
Age	50–64 (ref)	65.1	−14.7** (−18.8, −10.5)	−17.4** (−21.4, −13.5)	0.44** (0.37, 0.54)
	65 or over	50.4			
Gender	Male (ref)	56.5	8.2** (4.6, 11.7)	7.4** (4.0, 10.7)	1.41** (1.21, 1.65)
	Female	64.7			
Black Race	No (ref)	63.1	−13.3** (− 19.3, −7.3)	− 13.0** (− 18.5, −7.4)	0.55** (0.42, 0.71)
	Yes	49.8			
Hispanic Ethnicity	No (ref)	62.8	−11.6** (−18.0, −5.4)	−11.9** (− 17.7, −6.1)	0.57** (0.44, 0.75)
	Yes	51.2			
Married	No (ref)	62.5	−1.3 (−4.7, 2.0)	−0.4 (−3.8, 2.9)	0.98 (0.84, 1.14)
	Yes	61.2			
Graduated High School	No (ref)	52.8	11.1** (6.6, 15.6)	10.2** (5.8, 14.5)	1.61** (1.31, 1.97)
	Yes	63.9			
Employed	No (ref)	65.6	−10.2** (−13.7, −6.7)	−15.1** (− 18.8, − 11.4)	0.49** (0.41, 0.59)
	Yes	55.4			
Income Poverty	No (ref)	61.5	1.8 (−2.3, 5.8)	−1.4 (−5.8, 2.9)	0.94 (0.76, 1.15)
	Yes	63.3			
Health Insurance	No (ref)	51.6	11.5** (5.9, 17.1)	15.4** (10.3, 20.5)	2.05** (1.61, 2.62)
	Yes	63.1			
Substance Use Disorder	No (ref)	61.5	3.4 (−1.6, 8.4)	1.3 (−3.8, 6.4)	1.06 (0.84, 1.35)
	Yes	64.9			
Poor or Fair Self-Rated Health	No (ref)	61.1	1.7 (−1.7, 5.0)	−1.0 (−4.6, 2.5)	0.95 (0.81, 1.12)
	Yes	62.8			
Kessler-6 Score for Worst Month of the Past Year (continuous)		–	–	–	1.06** (1.05, 1.08)
Survey Year (continuous)		–	–	–	0.97 (0.93, 1.00)

* *p* < 0.01; ** *p* < 0.001

## Discussion

We found that the prevalence of SMMI was higher among those on parole or probation compared to all other older adults (21% vs. 7%, *p* <  0.001). Among older adults with SMMI, a higher proportion of those on parole or probation received any mental health treatment over the past 12 months compared to those who were not on correctional supervision (81% vs. 61%, *p* <  0.001). This association remained after adjusting for differences in sociodemographics and health. Older adults with SMMI and correctional supervision also had disproportionately high prevalence of co-occurring substance use disorders and socioeconomic disadvantage, which signals the need for complex health and social services coordination.

These results demonstrate that a large proportion (approximately 40%) of older adults with SMMI in the general population did not receive any mental health treatment, which is consistent with prior literature (Barry et al., 2012; Byers, Arean, & Yaffe, 2012; Han et al., 2011). Those on parole or probation were more likely to receive mental health treatment despite possessing several characteristics that are frequently associated with barriers to treatment in community-dwelling older adults, including male gender, socioeconomic disadvantage, and co-occurring substance use disorders (Barry et al., 2012; Garido, Kane, Kaas, & Kane, 2011; Han et al., 2011). While this study’s results do not include the precise elements of parole and probation that facilitated linkage to outpatient mental health treatment, we propose three potential explanations: mental health treatment delivered in prisons and jails; outpatient mental health care coordination in parole and probation settings; and court-mandated mental health and substance use treatment.

Mental health treatment delivered within prisons and jails could reduce stigma and enhance motivation for treatment. This explanation, if true, would counteract the widely held perception by clinicians and patients that mental health treatment is unacceptable or unnecessary for older adults (Alexopoulos, 2005; Callahan, Nienaber, Hendrie, & Tierney, 1992; Mackenzie, Pagura, & Sareen, 2010; Stewart, Jameson, & Curtin, 2015). Prior studies have shown that identification and treatment of mental health problems among incarcerated older adults can enhance coping resources (Maschi, Viola, Morgen, & Koskinen, 2015), which may improve resilience to stress and capacity to engage in outpatient treatment upon release from incarceration and reintegration into the community.

It is also possible that parole and probation services provide structured support and coordinating services for clients with mental illness, in turn reducing systemic barriers to mental health treatment access that are common among older adults (Brenes, Danhauer, Lyles, Hogan, & Miller, 2015). In addition to reducing stigma and enhancing motivation for treatment, parole and probation officers may help older adults overcome practical barriers related to cost, coverage, distance from services, and not knowing where to go. Best practices to coordinate healthcare and social services for individuals with mental illness during the reentry period emphasize cross-systems linkages, structured needs assessments, identification and engagement with existing community resources, education about the population for community providers, and development of targeted, evidence-based, and culturally competent interventions (Osher & King, 2015). The role of community corrections is often overlooked in these services coordination models, but vulnerable older adults with mental illness and few resources are likely to rely on corrections officers for support that goes beyond supervision and monitoring.

The third possible explanation for our findings is that high rates of mental health treatment in the community corrections group might reflect mandated court treatment for individuals diagnosed with mental illness and/or substance use disorders as part of an alternative sentencing scheme. Mental health courts, diversion programs, and specialized parole and probation services have emerged as interventions to address the “criminalization of mental illness,” i.e., the growing number of individuals with mental illness in the criminal justice system (Skeem, Manchak, & Peterson, 2011). Recent literature has questioned whether these programs reduce criminal recidivism, but they are still valuable to reduce mental health symptoms (Skeem et al., 2011). Since older justice-involved adults have lower recidivism rates than their younger counterparts (Piquero, Jennings, Diamond, & Reingle, 2015), programs that focus on mental health treatment linkage may be particularly important in this population.

The first two proposed explanations would suggest that comprehensive models of care, which have been developed to enhance service engagement for older adults with mental illness in the general population (Unutzer et al., 2002), are also capable of improving treatment rates among those on community correctional supervision. Existing reentry service models lack consideration of the unique needs of older adults, including geriatric syndromes, cognitive and functional impairment, and social role transitions specific to older adults (Metzger, Ahalt, Kushel, Riker, & Williams, 2017). Further studies on how to integrate evidence-based geriatric mental health interventions into existing reentry service coordination models are needed to tailor our community corrections programs to the needs of this growing population. The third proposed explanation, if true, might suggest that mental health courts, diversion programs, and specialized parole and probation services are particularly effective at facilitating mental health treatment for older adults on parole and probation.

Several of our secondary results also warrant additional exploration in future studies. For instance, individuals aged 65 or older and those without health insurance fared poorly in both groups (approximately 50% with SMMI received no treatment), whereas black individuals received treatment at much higher rates in the parole or probation group compared to those without community correctional supervision (85% vs. 48% received treatment). Further research is needed to replicate these preliminary findings and to shed light on the mechanisms responsible for the observed patterns.

Although older adults with SMMI were more likely to receive mental health treatment if they were on correctional supervision, nearly one out of every five individuals in this population (19%) received no treatment. This finding is worrisome, especially since the definition of “untreated” mental illness used in this study is quite severe: it requires no visits with any outpatient mental health providers and no mental health prescriptions, which is tantamount to no outpatient treatment whatsoever. The consequences of untreated or partially treated mental illness in older adults include poor quality of life, suicide, disability, cognitive impairment, greater likelihood of cardiovascular disease and chronic comorbidities, and economic loss (World Health Organization, 2003), all of which could be devastating in this already vulnerable population. Additional research is needed to understand barriers to healthcare access in this population and establish whether the treatment being received is appropriate and adequate to support positive mental health and criminal justice outcomes.

This study has several limitations. A relatively small sample size obviated our ability to use survey weights, which limits our ability to make population-based assumptions about our findings. Still, this is the first study to characterize outpatient mental health treatment among older adults on parole or probation with mental illness. Moreover, it is possible that the most disadvantaged and vulnerable older adults on parole or probation did not participate in the NSDUH, since the prevalence of justice involvement in the NSDUH sample lags behind national estimates (Kaeble, Maruschak, & Bonczar, 2015), and the NSDUH sampling frame is not well suited to identify individuals experiencing homelessness or other severe deprivation that is common in justice-involved populations (Williams et al., 2010). Therefore, our results could be interpreted as establishing a lower limit for population prevalence of mental illness among older adults on parole or probation. Finally, the NSDUH measures of mental illness are based on symptom severity rather than diagnosis, which means that individuals who lacked insight or whose symptoms were well controlled through treatment might not be identified as having SMMI. This could underestimate both the prevalence of diagnosable mental illness and the fraction of individuals with mental illness who received treatment. However, these results are still meaningful because self-reported symptom severity is an important person-centered mental health measure.

## Conclusions

A higher proportion of older adults on parole or probation received treatment for serious or moderate mental illness compared to those older adults with SMMI who were not on community correctional supervision. These findings suggest that community correctional supervising programs (parole and probation) are providing a critical coordinating role in linking older adults with mental illness to community mental health treatment. Nevertheless, the overall burden of mental health needs remained far higher in the population of older adults on community corrections. This study provides further evidence that linkage services between the criminal justice system and community-based care have the potential to increase and support access to mental health services for older adults. However, future research is needed to understand the best approaches to successful linkages, as well as the specific role that mental health services play in the complex services needs that older justice-involved individuals experience across mental health, physical health, substance use, and socioeconomic domains.

## Abbreviations

DSM-IV: Diagnostic and Statistical Manual of Mental Disorders, 4th Edition
GAF: Global Assessment of Functioning
K6: Kessler-6 Psychological Distress Scale
NIMH: National Institute of Mental Health
NSDUH: National Survey on Drug Use and Health
SAMHSA: Substance Abuse and Mental Health Services Administration
SMMI: Serious or Moderate Mental Illness
